# Association of Spanish as a Primary Language With Retear Rates After Pediatric ACL Reconstruction

**DOI:** 10.1177/23259671241252936

**Published:** 2024-06-13

**Authors:** Justin Lau, Edgar Garcia-Lopez, Brian T. Feeley, Nirav K. Pandya

**Affiliations:** * Department of Orthopaedic Surgery, University of California, San Francisco, San Francisco, California, USA; † San Francisco Veterans Affairs Health Care System, San Francisco, California, USA; Investigation performed at the Department of Orthopaedic Surgery, University of California, San Francisco, San Francisco, California USA

**Keywords:** primary spoken language, ACL reconstruction, outcomes, English vs Spanish, pediatrics

## Abstract

**Background:**

Anterior cruciate ligament (ACL) injuries are one of the most common knee injuries in pediatric patients in the United States. The patient's primary spoken language may affect outcomes after ACL reconstruction (ACLR).

**Purpose/Hypothesis:**

The purpose of this study was to identify differences in ACLR outcomes between patients whose primary, preferred spoken language was either English or Spanish. It was hypothesized that there would be a difference in retear rates between patients preferring English versus Spanish.

**Study Design:**

Cohort study; Level of evidence, 3.

**Methods:**

A retrospective cohort study was performed on pediatric and adolescent patients who underwent ACLR at a single institution. Patients were divided into 2 cohorts based on their preferred spoken language: English or Spanish. All patients underwent either hamstring tendon or bone–patellar tendon–bone autograft ACLR performed by the same surgeon with the same postoperative rehabilitation protocols. Linear regression, chi-square tests, and multivariate logistic regression were used to determine if outcomes, graft tear, revision surgery, and contralateral injury differed between groups.

**Results:**

A total of 68 patients were identified: 33 patients whose preferred language was English and 35 patients whose preferred language was Spanish. The overall mean age of the patients was 16.4 ± 1.4 years (range, 13.2-20.5 years), and the mean follow-up time was 3.26 ± 1.98 years (range, 0.53-8.13 years). Patients who preferred Spanish were more likely than those who preferred English to experience graft tears requiring revision surgery after ACLR (*P* = .02; odds ratio [OR] = 5.81; adjusted OR = 1.94), at a tear rate of 14.3%.

**Conclusion:**

Patients who preferred to speak Spanish experienced higher graft tear rates when compared with patients who preferred speaking English, even after adjusting for sex, sport played, graft type, type of insurance, and time to surgery.

Anterior cruciate ligament (ACL) injuries are one of the most common knee injuries in the United States,^19^ with an incidence rate of approximately 30 to 78 per 100,000 person-years.^10,11,19^ The incidence rate of ACL injury in children is even higher, with a mean incidence rate for patients aged 6 to 18 years of 121 ± 19 per 100,000 person-years from 1994 to 2013 and an annual increase in incidence rate of 2.3%.^3^ In conjunction with this rise in pediatric ACL injuries, Tepolt et al^21^ found a 5.7-fold increase in pediatric ACL reconstruction (ACLR) from 2004 to 2014. This trend is likely attributed to an increase of single sport specialization in adolescents at a younger age and increased participation in higher risk sports.^4,7,14,22^ These sports introduce many risk factors such as pivoting and shifting that produce rotational forces on the ligaments of the knee, resulting in ACL injuries.

In skeletally immature patients, ACLR holds additional risks compared with those in adults, such as increased risk of growth disturbance, high risk of graft tear based on graft choice, and risk of additional associated injuries.^2,20^ Multiple studies have evaluated the outcomes of ACLR centered on graft choices, drilling techniques, and postoperative rehabilitation protocols; however, factors such as race and ethnicity and socioeconomic status have had very limited attention in the literature.^6,13,15,16,23^

Prior studies have found worse outcomes in socially disadvantaged and publicly insured patients with ACL tears. Various studies have found that after ACL rupture, Black and Hispanic and publicly insured patients were more likely to experience delays in time evaluation, magnetic resonance imaging (MRI) to surgery, and return to play when compared with White and privately insured patients.^6,16,18^ These same patients had worse concurrent injuries, postoperatively averaged fewer physical therapy visits, and had significant postoperative reduction in strength and knee range of motion.^6^

With >20% of the US population >5 years of age being non-English speaking at home and >8% of the population having limited English proficiency, it is important to understand the impact of how primary spoken language affects postoperative outcomes, patient satisfaction, and quality of health care in orthopaedic subspecialties.^1,9^ Karliner et al^12^ found that patients with limited English proficiency were more likely to be readmitted following hospital admission than patients proficient in English, citing a lack of understanding of appointment time, medication, and discharge instructions. In a cohort of patients with a mean age of 47 years, Collins et al^8^ found that language influences the rates at which ACLR is performed, with patients preferring English being the most likely to undergo surgical reconstruction at 23%, followed by multilingual households at 16%, and patients who prefer to speak Spanish at 8%.^8^

There has been limited examination of long-term clinical care outcomes after ACLR centered around patient language preference. Without reporting of this data in the existing literature, clinicians may have a treatment “blind spot” that prevents them from understanding why adverse outcomes may be occurring irrespective of graft choice, surgical technique, or sporting activity. This also leads to an environment in which the development of interventions to mitigate these discrepancies is limited.

The purpose of this study was to examine the independent impact that a patient's primary language (English or Spanish) has on longitudinal surgical outcomes after ACLR. We hypothesized that there would be a difference in primary outcomes, as defined by retear and reoperation, between patients whose primary spoken language is Spanish compared with English.

## Methods

### Patient Selection

Institutional review board approval was obtained before data acquisition began and as this was a retrospective chart review without patient contact/intervention, no informed consent was deemed necessary by our IRB. This study was a retrospective review of pediatric surgical patients (defined as age <21 years by the US Food and Drug Administration; mean ± SD age, 16.4 ± 1.35 years; age range, 13.2-20.5 years) who underwent ACLR by the senior author (N.K.P.) at a single institution between 2016 and 2018. All patients underwent reconstruction using either hamstring tendon or bone–patellar tendon–bone autograft. From the senior author's surgical database, all patients who preferred speaking Spanish who met the inclusion criteria (n = 35) were identified as well as an appropriately matched cohort of patients preferring to speak English (n = 33) who underwent reconstruction immediately before or after (chronologically) the identified patients preferring Spanish. Both groups were matched according to age, sex, presence of meniscal tears, and high-risk sport participation. For each patient, having a primary, preferred language of Spanish was identified in the patient's electronic medical record and cross-referenced with the surgical consent, which indicated whether a certified translator was used. For patients aged <18 years, the primary language spoken by the parent/guardian was used. Patients were excluded from the study for the following reasons: <1 year of postoperative follow-up, indeterminate language status, surgery undertaken for congenital absence of the ACL, surgery performed for a multiligamentous knee injury, and/or the use of allograft as graft choice.

### Statistical Analysis

Patients were divided into 2 cohorts based on their primary, preferred spoken language: English or Spanish. The electronic medical record was searched to obtain demographics (age, sex), insurance type (public vs private), sport played (high-risk sports, defined as basketball, field hockey, football, hockey, lacrosse, rugby, soccer, and volleyball), intraoperative findings and associated procedures (graft choice/size, meniscal/cartilage surgery), postoperative adherence (defined as a lack of deviation from the postoperative protocol), and postoperative outcomes (complications, repeat surgery, graft tear).^5^ We quantified the relationship between a patient's preferred language and the variables noted above using linear regression analysis for continuous variables and the chi-square test for categorical variables.

To adjust for any potential confounding variables, multivariate logistic regression analysis was conducted to investigate the relationship between the dependent variable, graft tear, and independent variables including language, sex, high-risk sport, graft type, insurance type, and time to surgery. All demographic and surgical variables were initially included in the multivariate model even if nonsignificant when isolated, as their combination may result in significance. Adherence to the postoperative protocol was excluded from the multivariate analysis due to the multifaceted nature of being influenced by many variables that have not been accounted for in this study. Statistical analysis was performed using R statistical software (Version 4.3.1; R Foundation for Statistical Computing) with statistical significance set at *P* < .05.

Assuming a 9% ACL graft tear rate in the pediatric and adolescent population,^24^ a sample size of 34 patients per cohort (N = 68) was needed to detect a 28% difference in reinjury rates with an alpha of .05 and a power of 80%.

## Results

### Patient Demographics

**Figure 1.**
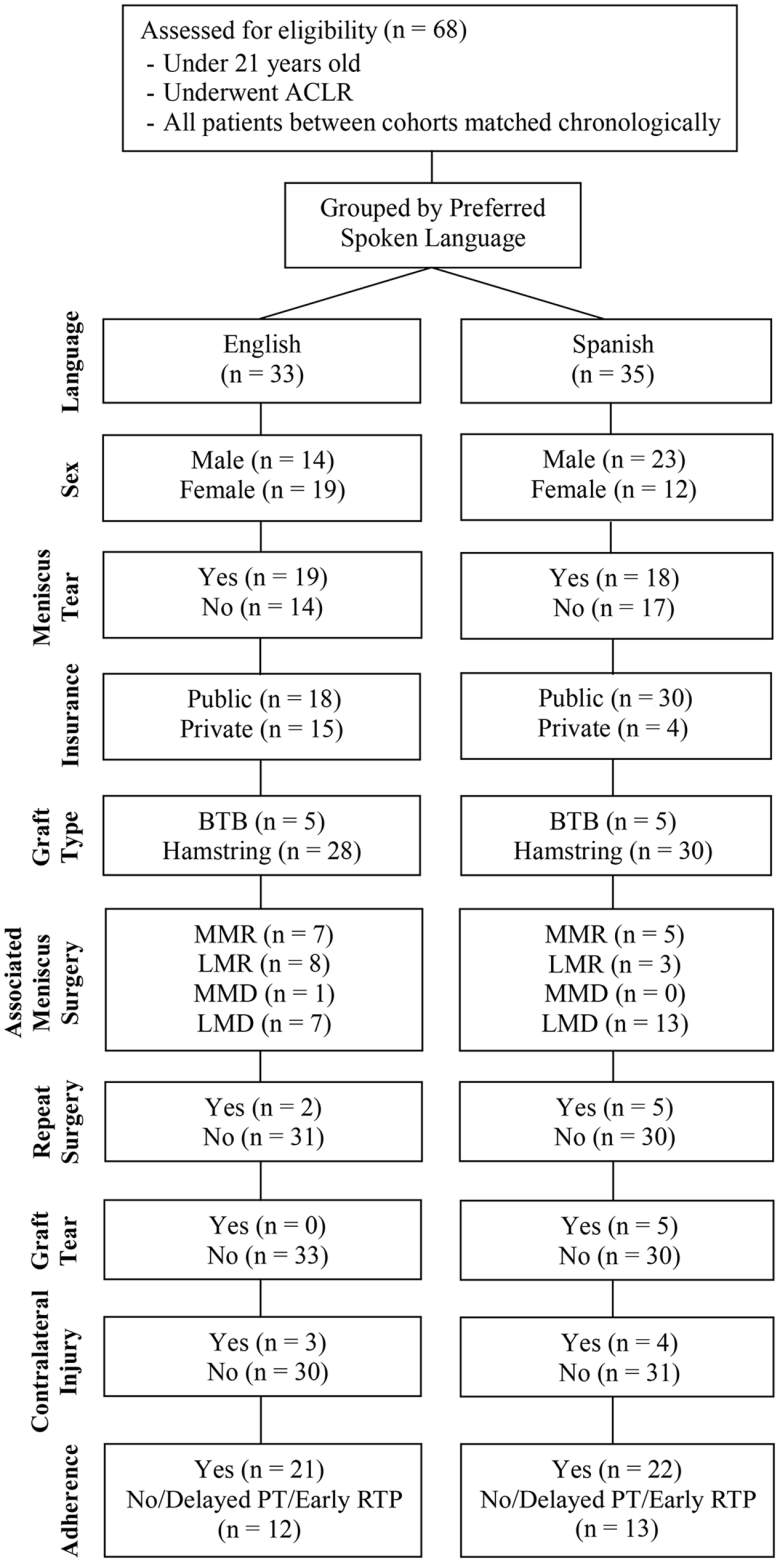
CONSORT (Consolidated Standards of Reporting Trials) flow diagram of the study groups. ACLR, anterior cruciate ligament reconstruction; BTB, bone-tendon-bone; LMD, lateral meniscal debridement; LMR, lateral meniscal repair; MMD, medial meniscal debridement; MMR, medial meniscal repair; PT, physical therapy; RTP, return to play.

Our cohort consisted of 33 patients preferring English and 35 preferring Spanish (Figure 1). The mean age of the patients preferring English was 16.3 ± 1.5 years (range, 13.6-20.5 years), while the patients preferring Spanish had a mean age of 16.4 ± 1.3 years (range, 13.2-18.2 years) (*P* = .74). The cohort who preferred English consisted of 14 male (42.4%) and 19 female patients (57.6%), while the cohort preferring Spanish consisted of 23 male (65.7%) and 12 female (34.3%) patients. The patient characteristics of both study groups are compared in Table 1. Overall, there were 37 (54%) patients with meniscal tears, with 19 patients (57.6%) in the cohort preferring English and 18 patients (51.4%) in the cohort preferring Spanish (*P* = .61). Of both cohorts, playing a high-risk sport was more common than not, 78.8% and 88.6% of the cohort preferring English and Spanish, respectively, with no significant group differences. All patients were insured, although insurance type for one of the patients preferring Spanish was not able to be identified. Significant group differences were found when comparing insurance type, with 54.5% of the cohort preferring English covered by a public medical insurance plan compared with 85.7% of the cohort preferring Spanish (*P* = .002).

**Table 1:** Patient Characteristics Between the Study Cohorts^*a*^

Variable	English Preferred (n = 33; 48.5%)	Spanish Preferred (n = 35; 51.5%)	*P*
Age, y, mean ± SD (range)	16.3 ± 1.46 (13.6-20.5)	16.42 ± 1.26 (13.2-18.8)	.74
Sex			.09
Male	14 (42.4)	23 (65.7)	
Female	19 (57.6)	12 (34.3)	
Meniscal tear			.61
Yes	19 (57.6)	18 (51.4)	
No	14 (42.4)	17 (48.6)	
High-risk sport			.44
Yes	26 (78.8)	31 (88.6)	
No	7 (21.2)	4 (11.4)	
Insurance type^*b*^			**.002**
Public	18 (54.5)	30 (85.7)	
Private	15 (45.5)	4 (11.4)	

a Data is reported as n (%) unless otherwise indicated. Boldface *P* value indicates statistically significant differences between groups (*P* < .05).

b The insurance type for 1 patient preferring Spanish was not able to be identified.

### Surgical Data

The comparison of surgical data between the study cohorts is detailed in Table 2. No significant group difference was found in the mean time from injury to surgery, which was 132.6 days (range, 9-564 days) for the English-preferred cohort versus 155.3 days (range, 22-620 days) for the Spanish-preferred cohort (*P* = .53). However, significant differences existed when comparing time to surgery versus the insurance type of the patient, with publicly insured patients typically waiting an additional 80 days before surgery than privately insured patients (*P* = .04). There were no group differences with respect to graft size, graft type, or associated meniscal surgery.

**Table 2:** Surgical Data Between the Study Groups^*a*^

Variable	English Preferred (n = 33; 48.5%)	Spanish Preferred (n = 35; 51.5%)	*P*
Time to surgery, d, mean ± SD (range)	132.6 ± 130 (9-564)	155.3 ± 151 (22-620)	.53
Graft size, mm, mean ± SD (range)	8.4 ± 0.61 (7.5-10)	8.2 ± 0.55 (7-9)	.19
Graft type			.92
BTB	5 (15.2)	5 (14.3)	
Hamstring	28 (84.8)	30 (85.7)	
Associated meniscal surgery			
Medial meniscal repair			.45
Yes	7 (21.2)	5 (14.3)	
No	26 (78.8)	30 (85.7)	
Lateral meniscal repair			.08
Yes	8 (24.2)	3 (8.6)	
No	25 (75.8)	32 (91.4)	
Medial meniscal debridement			.30
Yes	1 (3.0)	0 (0)	
No	32 (97.0)	35 (100)	
Lateral meniscal debridement			.15
Yes	7 (21.2)	13 (37.1)	
No	26 (78.8)	22 (62.9)	

a Data is reported as n (%) unless otherwise indicated. BTB, bone-tendon-bone.

### Postoperative Outcomes

Among the cohort preferring English, the mean follow-up time was 3.17 years, compared with 3.35 years in the cohort preferring Spanish (*P* = .71) (Table 3). There were no statistically significant differences between the 2 cohorts in terms of need for repeat surgery for reasons other than graft tear revision (eg, incision and drainage, manipulation under anesthesia, removal of hardware, and cyclops debridement) (*P* = .26). A higher percentage of the Spanish-preferred group experienced graft tears requiring revision surgery (14.3%) compared with the English-preferred group (0%) (*P* = .02), with an odds ratio (OR) of 5.81 (95% CI, 0.89-156.48) and an adjusted OR (after adjusting for sex, sport played, graft type, type of insurance, and time to surgery) of 1.94. Among the cohort preferring English, 3 out of 33 (9.1%) patients experienced contralateral injury, compared with 4 out of 35 (11.4%) of the patients preferring Spanish (*P* = .75). When we examined the graft tears as a model of graft choice (hamstring tendon/bone-tendon-bone), no statistically significant differences were identified between graft tears (*P* = .68).

**Table 3:** Postoperative Outcomes Between the Study Groups^*a*^

Variable	English Preferred (n = 33; 48.5%)	Spanish Preferred (n = 35; 51.5%)	*P*
Follow-up time, y, mean ± SD (range)	3.17 ± 2.01 (0.53-8.13)	3.35 ± 1.96 (0.58-7.90)	.71
Repeat surgery			.26
Yes	2 (6.1)	5 (14.3)	
No	31 (93.9)	30 (85.7)	
Graft tear requiring revision surgery			**.02**
Yes	0 (0)	5 (14.3)	
No	33 (100)	30 (85.7)	
Contralateral injury			.75
Yes	3 (9.1)	4 (11.4)	
No	30 (90.9)	31 (88.6)	
Adherence			.95
Yes	21 (63.6)	22 (62.9)	
No	3 (9.1)	1 (2.9)	.36
Delayed PT	7 (21.2)	8 (22.9)	.88
Early RTP	2 (6.1)	4 (11.4)	.48

a Data is reported as n (%) unless otherwise indicated. PT, physical therapy; RTP, return to play. Boldface *P* value indicates statistically significant differences between groups (*P* < .05).

Both groups experienced similar rates of adherence to postoperative protocols: 21 out of 33 patients preferring English (63.6%) and 22 out of 35 patients preferring Spanish (62.9%) (*P* = .95) (Table 3). When nonadherence rates were further analyzed by no compliance, delayed start of physical therapy (ie, >2 weeks postoperatively), and early return to play (<9 months postoperatively), no statistically significant differences were found between the 2 groups (*P* > .05).

### Results of Multivariate Analysis

When considering sex, high-risk sport, graft choice, insurance type, and time to surgery, significant differences still existed between the 2 cohorts in terms of graft tears requiring revision surgery (*R*^2^ = 20.3%; *P* = .04), with language being the significant predictor of the model (*P* = .03) (Table 4). Participation in a high-risk sport trended toward statistical significance (*P* = .05).

**Table 4:** Multivariate Analysis of Factors Predicting Graft Tears Requiring Revision Surgery^*a*^

Predictor	Estimate	SE	*T*	*P*
Intercept	0.13	0.14	0.90	.37
Language	0.15	0.072	2.15	**.03**
Sex	0.062	0.073	0.85	.40
High-risk sport	−0.18	0.090	−2.02	.05
Graft type	0.081	0.092	0.88	.38
Insurance (public vs private)	−0.050	0.085	−0.58	.56
Time to surgery	−0.00047	0.00025	−1.87	.07

a Boldface *P* value indicates statistically significant differences between groups (*P* < .05).

## Discussion

The results of this study demonstrate that in a cohort of pediatric patients who underwent ACLR by the same surgeon (N.K.P.), language spoken was associated with postoperative outcomes. Even after adjusting for demographics, graft choice, and insurance type, significant differences in outcome still existed between the study cohorts, with patients preferring Spanish having a higher incidence of graft tears (OR = 5.81; adjusted OR = 1.94) when compared with patients preferring English.

As language was the only statistically significant predictor of graft retear within the multivariate model in the current study, it had the highest overall contribution to the prediction of graft tears between the 2 cohorts. In a retrospective study comparing adult patient demographics associated with ACL surgery, Collins et al^8^ found that patients who preferred Spanish were less likely to undergo ACLR when compared with patients preferring English (8% vs 23%).^8^ Similar trends were observed in patients with lower socioeconomic status and public insurance. Similarly in this study, graft tears requiring revision surgery occurred in patients who were covered by public insurance. However, patients in the cohort speaking Spanish were still more likely to experience a graft tear requiring revision surgery even after adjusting for their insurance as well as delayed time to surgery. This indicates a need to examine the relationship between the preferred language of the patient, especially when non-English, and its effect on postoperative outcomes and highlights the importance of investigating the known relationships between language, socioeconomic status, race, and insurance on postoperative outcomes.^6,15,16,23^

In our study, patients preferring Spanish were found to be more likely to have public insurance. Prior studies have found that patients with public insurance tend to perform worse postoperatively when compared with their privately insured counterparts.^6,16^ In a study evaluating the effect of insurance and socioeconomic status on post-ACLR outcomes in pediatric patients, Patel et al^16^ found publicly insured patients to have a longer time between injury and return to play and higher incidence of postoperative stiffness. Interestingly, Bram et al^6^ found that privately insured patients were more likely to experience graft tear than publicly insured patients. This differed from the finding of this study, and the underlying cause of such discrepancy needs to be examined to properly understand the discrepancy of care that exists between the 2 cohorts. To understand the causes of postoperative outcome discrepancies, such as in the incidence of graft tears, that exist between patients who prefer speaking either English or Spanish, it is thus important to also determine the role insurance plays on outcome in the context of spoken language.

In this study, no differences were found between patients preferring English or Spanish in terms of time to surgery (*P* = .53). No prior studies have examined the effect of language on time to surgery; however, other studies have looked at the effect of insurance type on time to ACLR.^6,16,23^ A study of a similar cohort of patients who underwent ACLR and were managed by the same surgeon found that the time from injury to MRI diagnosis and surgical treatment was significantly shorter for patients who were privately insured as opposed to publicly insured.^13^ Three other studies found that publicly insured pediatric patients were evaluated on average 136 days after injury in comparison with 56 days for those who were privately insured.^6,16,23^ Publicly insured, racial and ethnic minority, and lower socioeconomic status pediatric patients also had longer time to MRI and surgery, resulting in increased likelihood of irreparable meniscus tears and chondral lesions ≥grade 2.^6,16,23^ Bram et al^6^ found that publicly insured and racial and ethnic minority patients had fewer physical therapy visits and exhibited greater strength and range of motion deficits in terms of pediatric post-ACLR outcomes. This may be due to rehabilitation facilities’ acceptance of insurance: in 1 study,^17^ rehabilitation facilities were more likely to accept private insurance (96.4%) when compared with public insurance (51.8%). Although this study found no differences in time to surgery between the 2 groups, patients preferring Spanish were found to be more likely to be covered by public insurance (*P* = .002). Understanding the relationship between the primary spoken language of the patient and his or her insurance will provide further insight on discrepancies in care that result in increased time to surgery and other deficits in postoperative outcomes.

While adherence to the heavily detailed post-ACLR protocol may be cited for the discrepancy in outcome, no statistically significant differences were found in terms of adherence to the postoperative protocol (general noncompliance, delayed physical therapy, and early return to play) between patients preferring English or Spanish. However, there may still be some aspect of verbal communication/language within points of postoperative care that influences successful outcome. Although the physical therapy establishments used by the patients in this study had access to interpreters, there may be barriers other than language preventing progression in post-operative care for patients preferring Spanish that needs to be further examined.

The etiology of the results observed in this study from a language perspective is likely multifaceted. With graft tear rates remaining significantly different between the 2 cohorts even when adjusting for demographics, graft choice, and insurance type, it is likely there is some linguistic barrier between patient and provider that works with other demographic factors to predict postoperative outcomes. As such, additional research needs to be performed to rectify such language-based differences after ACLR. The first step to understanding the relationship between language and outcome is to fully understand the ways language, insurance, socioeconomic status, and the postoperative protocol work together to influence outcome as well as the extent to which spoken language alone affects postoperative outcomes. This will allow for a better understanding of where language-specific discrepancies exist within the longitudinal care pathway of the patient who undergoes surgery, starting from the time of injury, both within the office and at touch points outside of the office including telephone calls, electronic communication (or lack thereof), physical therapy visits, athletic trainers, and coaches during the return to play process as compared with discrepancies that could be attributed to socioeconomic status or insurance type. The second step is to develop interventions that can help to rectify these differences and to then study their efficacy. This goes beyond simply providing an interpreter for the surgical consent process and in-office visits to also providing Spanish-language materials that are extend beyond the exam room such as educational handouts, after-visit summaries, postoperative protocols, home exercise programs, and videos. Identification of health care providers who speak Spanish in the physical therapy and school (both coaches and athletic trainers) settings are critical as well.

### Limitations

There are several limitations in our study. First, the modest sample size of the study in a single institution by a single provider limited the ability of our research to be generalized to other practice settings and geographic locations. Second, the patients’ socioeconomic status was not considered with regard to the data that were analyzed. This demographic is an important factor to consider when it comes to understanding discrepancies in care and could help provide added information regarding adherence to the postoperative protocol and other factors that would affect postoperative outcomes. Third, as this was a retrospective study, reliable data were not available in the medical record as to how often an interpreter was used in the care setting beyond the surgical consent process. Understanding how and to what extent an interpreter was used could be helpful to fully understand the linguistic barrier that existed between provider and patient. Fourth, follow-up was limited to 1 year, as patients were discharged after that period based on hospital protocol. Longer follow-up time points would be helpful to better determine outcome and identify the discrepancies that exist. Fifth, this study was performed in a pediatric and adolescent cohort of patients. A study that was performed in an adult population may render different findings from those of our cohort. A retrospective study cannot prove cause and effect. Future prospective research is needed to further examine language-based discrepancies in a large population in order to provide a model through which interventions can be developed. Finally, the importance of patient reported outcomes has been highlighted in the literature but was not assessed in this study.

## Conclusion

In a practice where pediatric patients received ACLR by the same surgeon and were assigned the same postoperative protocol, patients preferring Spanish were more likely to experience a graft tear requiring revision surgery when compared with patients preferring English even when controlling for other demographic variables such as insurance type. Future research needs to be performed that identifies points in the care pathway where language-based interventions can be introduced to improve postoperative outcomes, followed by studies examining the efficacy of these language-based interventions.
